# Age-related methylation changes in the human sperm epigenome

**DOI:** 10.18632/aging.204546

**Published:** 2023-02-27

**Authors:** Laura Bernhardt, Marcus Dittrich, Andreas Prell, Ramya Potabattula, Charis Drummer, Rüdiger Behr, Thomas Hahn, Martin Schorsch, Tobias Müller, Thomas Haaf

**Affiliations:** 1 Institute of Human Genetics, Julius Maximilians University, Würzburg, Germany; 2 Department of Bioinformatics, Julius Maximilians University, Würzburg, Germany; 3 Platform Degenerative Diseases, German Primate Center, Leibniz Institute for Primate Research, Göttingen and German Center for Cardiovascular Research, Partner Site Göttingen, Göttingen, Germany; 4 Fertility Center, Wiesbaden, Germany

**Keywords:** ART outcome, DNA methylation, male germ cells, paternal age effect, human sperm epigenome

## Abstract

Advanced paternal age is associated with increased risks for reproductive and offspring medical problems. Accumulating evidence suggests age-related changes in the sperm epigenome as one underlying mechanism. Using reduced representation bisulfite sequencing on 73 sperm samples of males attending a fertility center, we identified 1,162 (74%) regions which were significantly (FDR-adjusted) hypomethylated and 403 regions (26%) being hypermethylated with age. There were no significant correlations with paternal BMI, semen quality, or ART outcome. The majority (1,152 of 1,565; 74%) of age-related differentially methylated regions (ageDMRs) were located within genic regions, including 1,002 genes with symbols. Hypomethylated ageDMRs were closer to transcription start sites than hypermethylated DMRs, half of which reside in gene-distal regions. In this and conceptually related genome-wide studies, so far 2,355 genes have been reported with significant sperm ageDMRs, however most (90%) of them in only one study. The 241 genes which have been replicated at least once showed significant functional enrichments in 41 biological processes associated with development and the nervous system and in 10 cellular components associated with synapses and neurons. This supports the hypothesis that paternal age effects on the sperm methylome affect offspring behaviour and neurodevelopment. It is interesting to note that sperm ageDMRs were not randomly distributed throughout the human genome; chromosome 19 showed a highly significant twofold enrichment with sperm ageDMRs. Although the high gene density and CpG content have been conserved, the orthologous marmoset chromosome 22 did not appear to exhibit an increased regulatory potential by age-related DNA methylation changes.

## INTRODUCTION

Despite decreasing reproductive success and adverse health outcomes of the offspring, the trend towards delayed parenthood has been constantly increasing over the past decades. For economic, social, political, and cultural reasons many parents postpone offspring conception beyond the optimal biological age [1], increasing the demand for assisted reproductive technologies (ARTs) and prenatal diagnostic testing. For a long time, medical problems associated with advanced parental age were primarily attributed to maternal aging. The decline in ovarian reserve and prolonged meiotic arrest, associated with an increased oocyte aneuploidy rate, can cause fertility problems, miscarriages, and children with Down syndrome and other aneuploidies [2, 3].

However, the chances of aging men to achieve a pregnancy is also reduced [4–6]. This appears to be due to declining sperm quality rather than quantity. Higher paternal age increases the offspring’s risk for some rare monogenic disorders due to de-novo genetic mutations [7] as well as for complex neurodevelopmental disorders, including attention deficit disorder, autism spectrum disorder, and schizophrenia [8]. In addition, advanced paternal age is associated with subtle impaired neurocognitive outcomes during infancy and childhood [9]. Oocytes are meiotically arrested in the fetal germline, whereas the number of spermatogonial cell divisions in the continuously dividing male germline increases from 35 times at puberty to > 800 times at the age of 50 years [7]. During each replication cycle, not only the DNA sequence itself, but also its epigenetic marks must be correctly copied to the daughter cells. Since the error rate of this copying process is at least one order of magnitude higher for epigenetic than for genetic information [10], the spermatozoa from older males are endowed with many more epigenetic than DNA sequence changes.

The sperm epigenome is the end product of male germline reprogramming and is fundamentally different from the epigenomes of oocytes and somatic cells [11]. Moreover, it is affected by stochastic and environmental factors, including fertility status, diet, and aging [12–14]. Different techniques including Illumina methylation arrays [15–18], reduced representation bisulfite sequencing (RRBS) [19], whole genome bisulfite sequencing (WGBS) [20], and methylcytosine capture sequencing [21] have been used to study age-related changes in the human sperm methylome. Several epigenetic clocks were derived by linear regression algorithms on different methylation data sets for human sperm age prediction [14, 17, 20–22]. Although in general there is little overlap between the age-related differentially methylated regions (ageDMRs) in different studies, the methylation changes appear to be enriched in genes associated with embryonic and neuronal development [16, 18–20].

It is tempting to speculate that age-associated methylation changes in sperm and their impact on gene regulation are transmitted to the next generation, contributing to developmental competence of the resulting embryos and health and disease of the offspring. Denomme et al. [19] reported correlations between age-related alterations in human sperm (identified by RRBS) and blastocysts (identified by WGBS). In the aging mouse model, sperm DNA methylation changes have been associated with changes in gene methylation and expression in the brain and abnormal behavior in the offspring derived from older males [23].

## RESULTS

### Clinical parameters

RRBS was performed on 73 sperm samples from couples undergoing infertility treatment by IVF and/or ICSI (Supplementary Table 1). Most (56 of 73, 77%) samples had a concentration higher than 15 x 10^6^/ml, a total motility of more than 40%, and more than 4% sperm with normal morphology. Normal semen parameters according to the 5^th^ edition of the WHO laboratory manual [24] were considered as an indicator for male fertility potential. A subgroup (17 of 73; 23%) of samples had abnormal spermiograms, indicative of reduced fertility potential. The age of the sperm donors ranged from 25.8 to 50.4 years, the body mass index (BMI) from 17.5 to 37.8 kg/m^2^. A pregnancy was achieved with 42 of these samples, whereas 30 samples did not yield a pregnancy. Interestingly, the pregnancy rate was somewhat higher with samples showing abnormal spermiograms (11 of 17; 64%) than with normozoospermic samples (31 of 56; 55%). This may reflect the predominant usage of ICSI (13 of 17; 77%) in the subfertile group and of IVF (49 of 56; 87%) in the normozoospermic group. The factors "age", "BMI", "semen parameters (normal/abnormal)", and "pregnancy outcome (yes/no)" were considered in a model to study effects on sperm methylation.

### Normal methylation imprints in IVF/ICSI sperm samples

To exclude somatic cell contamination, we selected 8 maternally methylated and three paternally methylated imprinting control regions (ICRs) that were well covered in our RRBS data set from a list of 50 known human germline DMRs [25, 26]. As expected, all analyzed oocyte germline DMRs, GNAS A/B:TSS-DMR (median methylation 0.4%; range 0-3%), GNAS-XL:Ex1-DMR (0.4%; 0.1-2.3%), GNAS-AS1:TSS-DMR (0.4%; 0-3.5%), GNAS-NESP:TSS-DMR (0.4%; 0.1-2.9%), SNURF:TSS-DMR (0.4%; 0-2.6%), SNRPN:Int1-DMR2 (0%; 0-7.6%), KCNQ1OT1:TSS-DMR (0.4%; 0.1-3.8%), and MEST:alt-TSS-DMR (0.4%; 0-2.7%) were strongly hypomethylated in sperm (Supplementary Figure 1). Not a single sample displayed a methylation value ≥ 10%. The three analysed sperm germline DMRs, IGF2:alt-TSS-DMR (97%; 89-100%), IGF2:Ex9-DMR (95%; 90-98%), and H19/IGF2:IG-DMR (83%; 71-93%) were hypermethylated in sperm. Compared to other DMRs, the H19/IGF2:IG-DMR showed a somewhat larger methylation variation, however all methylation values were consistent with correct paternal imprinting. Thus, none of our 73 study samples displayed an abnormally (de)methylated ICR in any of the 11 studied imprinted genes.

### Sperm age DMRs

**Figure 1:**
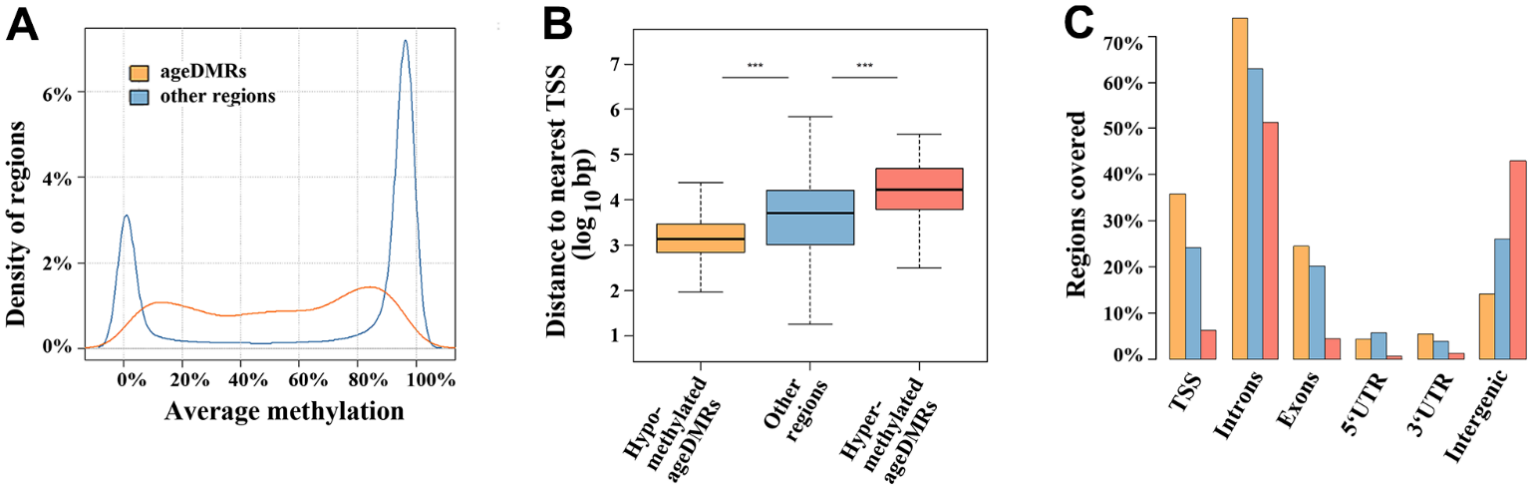
**Region characteristics.** (**A**) Distribution of methylation levels of ageDMRs versus other (non-significant) regions. The average methylation levels of ageDMRs (orange line) are predominantly in the mid-range (20-80%), whereas other regions (blue line) are either in the low range (< 20%) or high range (> 80%) of methylation. (**B**) Box plots showing the distance of analyzed regions to the nearest transcription start site (TSS). The median is represented by a horizontal line. The bottom of the box indicates the 25^th^ percentile, the top the 75^th^ percentile. The blue box represents non-significant regions, the orange box ageDMRs which lose methylation with age and the red box ageDMRs which gain methylation with age. Please note that hypomethylated ageDMRs are significantly (*** *P* < 0.001) closer to the TSS, whereas hypermethylated DMRs are more distant from the TSS than other regions. (**C**) Localization of hypomethlyated ageDMRs (orange bars) and hypermethylated DMRs (red bars) in different genic and intergenic regions, compared to non-significant regions (blue bars). Please note that some regions may be assigned to several gene parts and, therefore, the percentages of all bars (of one color) total > 100%.

Out of 360,264 analysed regions, 1,565 (0.4%) showed a significant (FDR-adjusted) correlation between sperm methylation and donor age (Supplementary Table 2). The mean DMR length (± standard deviation, SD) was 523 ± 297 bp. The direction of age association was highly skewed with 1,162 (74%) ageDMRs being hypomethylated and 403 (26%) being hypermethylated with age. Interestingly, we did not detect genome-wide significant DMRs associated with BMI, semen quality, and pregnancy outcome. The majority (836 of 1,565; 53%) of ageDMRs displayed an average methylation in the medium range (20-80%); 22% were in the low range (< 20%) and 25% in the high range (> 80%) of methylation (Figure 1A). In contrast, most (228,186 of 358,699; 64%) other regions not subject to paternal age effects were in the high, 30% in the low, and only 9% in the medium range of methylation. Hypomethylated ageDMRs were closer to the nearest transcription start site (median distance to TSS 1,368 bp, [IQR 709, 2,910], *P* < 0.001), compared to other regions (median 5,202 [IQR 1,066, 16,260]), whereas hypermethylated ageDMRs (median 17,205 bp [IQR 6,381, 49,512], *P* < 0.001) were located more distantly from the nearest TSS (Figure 1B). Hypomethylated ageDMRs were preferentially located around TSS, in exons and introns, whereas hypermethylated DMRs were underrepresented in TSS and enriched in intergenic regions (Figure 1C).

**Figure 2:**
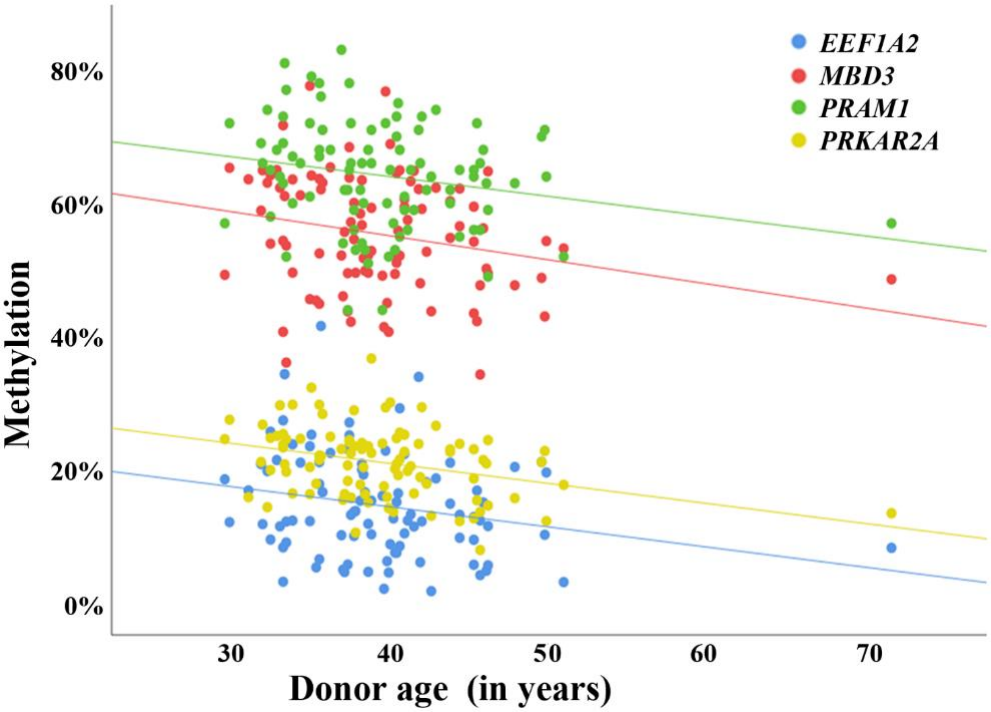
**Validation of sperm ageDMRs by bisulfite pyrosequencing.** Scatter plots showing the correlations between average regional methylation (y-axis in %), determined by bisulfite pyrosequencing, and donor age (x-axis in years) in 94 human sperm samples. The blue dots represent pyrosequencing measurements for an ageDMR (identified by RRBS) in *EEF1A2*, the red dots for an ageDMR in *MBD3*, the green dots for an ageDMR in *PRAM1*, and the yellow dots for an ageDMR in *PRKAR2A*. Consistent with the results of RRBS, the regression lines of all analyzed regions indicate a significant (also see Supplementary Table 3) loss of methylation with age. The correlations remain virtually unchanged when excluding the 72-year-old sample from analysis.

To demonstrate the validity of our data set, four genes were randomly picked from our list of 1,565 sperm ageDMRs, namely *PRAM1* (no. 59 in Supplementary Table 2), *EEF1A2* (no. 183), *PRKAR2A* (no. 1,068), and *MBD3* (no. 1,158). Consistent with RRBS, bisulfite pyrosequencing of 94 independent sperm samples showed a significant age-related decrease of methylation for all four genes (Figure 2 and Supplementary Table 3).

Using the Genomic Regions Enrichment of Annotations Tool (GREAT), hypomethylated sperm ageDMRs were enriched in human/mouse phenotypes related to male infertility (Table 1). In contrast, hypermethylated ageDMRs were overrepresented in biological processes associated with synapses and in phenotypes associated with abnormal behaviour.

**Table 1:** Functional enrichment analysis of sperm ageDMRs.

**Hypomethylated sperm ageDMRs**	**FDR Q value**	**Fold enrichment**
**Biological processes**		
Progesterone biosynthetic process	2.7E-02	24.6
Peptidyl-lysine modification to peptidyl-hypusine	1.5E-02	24.2
**Molecular function**		
Deoxyhypusine monooxygenase activity	4.4E-04	44.3
Thioredoxin peroxidase activity	2.6E-02	17.2
**Human phenotypes**		
Testicular microlithiasis	2.8E-02	15.4
Decreased serum testosterone level	6.3E-03	21.8
Decreased circulating luteinizing hormone level	4.1E-04	39.8
Androgen insufficiency	4.5E-05	73.8
Abnormality of the Leydig cells	4.5E-05	73.8
Abnormal circulating luteinizing hormone level	4.6E-02	9.7
**Mouse single KO phenotype**		
Small bulbourethral gland	6.6E-02	19.1
Leydig cell hypoplasia	3.7E-02	5.5
**Hypermethylated sperm ageDMRs**	**FDR Q value**	**Fold enrichment**
**Biological processes**		
Homophilic cell adhesion via plasma membrane adhesion molecules	2.8E-04	3.6
Cell-cell adhesion via plasma-membrane adhesion molecules	2.2E-04	3.1
Positive regulation of synapse assembly	3.3E-04	4.3
Synapse assembly	3.5E-04	4.4
Regulation of synapse assembly	3.5E-04	3.8
Regulation of synapse organization	3.4E-02	3.1
Regulation of synapse structure or activity	4.2E-03	3.0
**Human phenotypes**		
Hair-pulling	9.6E-08	69.5
Phonic tics	9.6E-08	69.5
Echolalia	1.5E-06	28.4
Tics	7.4E-06	22.2
Motor tics	6.3E-06	30.5
Self-mutilation	6.6E-03	7.2
Multifactorial inheritance	1.8E-02	7.3
Obsessive-compulsive behavior	3.1E-02	7.9
Self-injurious behavior	3.2E-02	5.6
**Mouse single KO phenotypes**		
Decreased coping response	1.4E-04	14.9
Abnormal noradrenaline level	3.4E-03	5.3
Abnormal coping response	3.1E-03	5.1
Abnormal vestibular system physiology	1.1E-02	5.2
Increased fear-related response	1.6E-02	9.9
Abnormal depression-related behavior	2.7E-02	3.1
Increased circulating thyroxine level	3.0E-02	10.9
Enlarged epididymis	3.1E-02	6.0
Abnormal vestibuloocular reflex	3.0E-02	14.5

### Comparison of our set of sperm ageDMR-associated genes with published data sets

In our analysis, 1,152 out of 1,565 significant sperm ageDMRs are located within genic regions, amounting to a total of 1,002 different gene symbols that are associated with at least one sperm ageDMR. This number is considerably higher than in most conceptually related studies that have investigated paternal age effects on the sperm epigenome (Supplementary Figure 2) [15–21]. Interestingly, six (*GNG7*, *MAU2*, *PPP2R3A*, *PPP5D1P*, *SUZ12P1*, and *USP34*) of the > 100 genes with two or three ageDMRs were endowed with both hypomethylated and hypermethylated ageDMRs in different genic regions (Supplementary Table 2).

To further corroborate our candidate genes, we collected the lists of gene symbols with sperm ageDMRs from seven published studies. Meta-analysis genes have been counted as matching if they have been reported as ageDMR gene regardless of the direction of methylation change. Including our data set, we obtained a set of 2,355 genes with evidence of age-related methylation changes (Supplementary Table 4). The majority (2,116; 90%) of these genes have been reported in only one study (Supplementary Figure 2), whereas 241 genes have been replicated at least once. Three genes displayed genome-wide significant sperm ageDMRs in 5 studies, 6 in four, 29 in three, and 203 in two studies (Table 2).

**Table 2:** Genes with sperm ageDMRs in independent genome-wide studies.

Number of studies*	**Genes with sperm ageDMRs**
5	*DLGAP2*, *PRDM16*, and *SLC22A18AS*
4	*C7orf5*, *KCNQX*, *NCOR2*, *THBS3*, *TNXB*, and *UTS2R*
3	*ADARB2, ANOX, ARID3C, BCLXXA, BEGAIN, CRYBA2, DLLX, DMPK, FAM86JP, FBXO2, FGF8, GET4, GPANKX, GRINX, KCNA7, LMO3, NSGX, NTM, PAX2, PCDHX5, PINXX, PURA, PYY2, SECTMX, SEMA6B, SOHLHX, STRA8, TTC7B,* and *WDR27*
2	*ABCA7, ABLIMX, ADAM33, ADAMTSX6, ADAMTS8, ADRB3, AIM2, AJAPX, ANK2, ANKRDX, ANKSXB, ANP32APX, ARC, ARHGAP39, ARHGEFX, ARMC3, ARPP2X, ASBX8, ATHLX, ATNX, ATXN7L3, B4GALNTX, B4GALNT4, BARHLX, BLCAP, BMP8A, CXorf86, CABSX, CALCA, CCDCXX4, CCDCX44NL, CCDCX82, CCR6, CDHX3, CDHX8, CDH22, CFD, CHMPXA, CHRNE, CHST8, CKAP4, CLICX, COL23AX, COLGALTX, CRLFX, CROCC, DAPK3, DHRSX, DHXX6, DLKX, DNMTX, DOHH, DOK2, DSEL-ASX, DVLX, EEFXA2, EFCAB4A, EGFL7, EHMTX, EPDRX, EPHAX, EPHB4, EPN2, EPS8LX, EVXX, EXPH5, FAM86CX, FBN3, FBRSLX, FGF3, FOXF2, FOXKX, FSCNX, GABRB3, GALNT9, GAPDH, GATA2, GNB2, GNG7, GPCX, GPERX, GPRX5X, GPR45, GPT2, HMX3, HOXAX, HOXB6, HOXDX, HYAL2, IGFX, IGF2, IGSFX, IGSF2X, INSRR, JAM3, KCNIP4, KCNQ2, KDM2B, KDM4B, KRTX9, KRT4, LAMA2, LCK, LDLRAD4, LHXX, LHX3, LINC2X82, LINGOX, LMNB2, LOCXX3346X, LONPX, LRCH4, LRFN2, MACRODX, MALRDX, MAP3KX, MAPK8IP2, MCTP2, METRNL, MIR22HG, MIR9-3, MNX, MRPL36, MTMR8, MUCX, NADK, NAPXL4, NCDN, NINJ2, NPL, NR4A2, NSMF, NSUN5, NXPH4, NYAP2, PAX3, PAX6, PCDHX7, PCGF3, PCLO, PCOLCE, PDLIM3, PITXX, PLK5, PPFIA2, PPPXRX8, PPPXR27, PPP2R2C, PPP2R2D, PPP2R5B, PPP2R5E, PRKCZ, PRRC2A, PRSS22, PSMB8, PTPRS, RASA3, RBFOXX, RPLX3, RUBCN, RYR2, SAP25, SDCCAG8, SEMA3A, SETBPX, SEZ6, SHANK2, SLCX4A2, SLC24A5, SLC26AX, SLC4A2, SLC8A2, SNHGX, SNTG2, SORBS2, SRSF5, SRSF7, SSTR5, ST5, STOX2, STX2, SYNE4, TBKBPX, TBX5, TCL6, TENM3, TFEB, THBS2, TIMMX3, TIMM44, TINAG, TMEMX32D, TMEM59L, TNK2, TP73, TUBB, UNKL, XKR6, YPEL4, ZFHX3, ZNF5X6, ZNF536,* and *ZNF853*

*Including this study, Jenkins et al. [16, 17], Lee et al. [15], Cao et al. [21], Denomme et al. [19], Laurentino et al. [20], and Oluwayiose et al. [18].

To investigate whether the different studies hold a common signal, we performed an enrichment analysis comparing our sperm ageDMR-associated genes with the reported gene sets. For each of the seven included studies, we found a significant enrichment of published genes among our set of genes with sperm age DMRs (Supplementary Table 5), supporting the existence of a common effect of paternal age on sperm methylation. In functional enrichment analysis, the 241 replicated genes were significantly (adjusted *P* ≤ 0.015) overrepresented in 44 biological processes, many of them associated with development (18 terms), neurodevelopment (16 terms), and synapses (7 terms) as well as in 18 cellular components, associated with synapses (7 terms) and neurons (3 terms). Moreover, the input genes were significantly (adj. *P* ≤ 0.015) enriched with 26 transcription factor binding motifs (Table 3).

**Table 3:** Enrichment analysis of 241 replicated genes with sperm ageDMRs.

**Biological processes**	**Term ID**	**Adjusted *P***
Developmental process	GO:0032502	1.14E-07
System development	GO:0048731	4.47E-06
Anatomical structure development	GO:0048856	8.23E-06
Multicellular organism development	GO:0007275	1.43E-07
Nervous system development	GO:0007399	2.01E-09
Multicellular organismal process	GO:0032501	3.5E-07
Anatomical structure morphogenesis	GO:0009653	2.1E-05
Animal organ morphogenesis	GO:0009887	3.3E-04
Chemical synaptic transmission	GO:0007268	3.7E-04
Anterograde trans-synaptic signaling	GO:0098916	3.7E-04
Trans-synaptic signaling	GO:0099537	4.5E-04
Modulation of chemical synaptic transmission	GO:0050804	5.2E-04
Regulation of transsynaptic signaling	GO:0099177	5.4E-04
Cell differentiation	GO:0030154	6.5E-04
Synaptic signaling	GO:0099536	8.3E-04
Neuron development	GO:0048666	8.8E-04
Cellular component morphogenesis	GO:0032989	9.3E-04
Embryonic organ morphogenesis	GO:0048562	1.3E-03
Animal organ development	GO:0048513	1.4E-03
Cellular developmental process	GO:0048869	1.5E-03
Cell morphogenesis involved in neuron differentiation	GO:0048667	2.2E-03
Neurogenesis	GO:0022008	2.3E-03
Neuron projection morphogenesis	GO:0048812	2.5E-03
Cell junction organization	GO:0034330	3.1E-03
Generation of neurons	GO:0048699	3.2E-03
Embryonic organ development	GO:0048568	3.3E-03
Plasma membrane bounded cell projection morphogenesis	GO:0120039	3.6E-03
Cell projection morphogenesis	GO:0048858	4.0E-03
Obsolete gene silencing	GO:0016458	4.4E-03
Plasma membrane bounded cell projection organization	GO:0120036	4.9E-03
Neuron differentiation	GO:0030182	5.3E-03
Embryo development	GO:0009790	5.5E-03
Regulation of cellular process	GO:0050794	6.0E-03
Neuron migration	GO:0001764	6.1E-03
Neuron projection development	GO:0031175	6.1E-03
Cell part morphogenesis	GO:0032990	6.2E-03
Cell-cell signaling	GO:0007267	8.0E-03
Cell projection organization	GO:0030030	9.0E-03
Muscle structure development	GO:0061061	9.0E-03
Excitatory postsynaptic potential	GO:0060079	9.1E-03
Regulation of cell communication	GO:0010646	0.010
Regulation of signaling	GO:0023051	0.012
Diencephalon development	GO:0021536	0.012
Positive regulation of transcription by RNA polymerase II	GO:0045944	0.012
**Cellular component**	**Term ID**	**Adjusted *P***
Postsynapse	GO:0098794	2.5E-08
Cell junction	GO:0030054	1.2E-06
Synapse	GO:0045202	1.4E-05
Neuron projection	GO:0043005	5.2E-04
Postsynaptic density	GO:0014069	7.8E-04
Plasma membrane bounded cell projection	GO:0120025	8.1E-04
Asymmetric synapse	GO:0032279	9.6E-04
Postsynaptic specialization	GO:0099572	1.8E-03
Cell periphery	GO:0071944	1.9E-03
Neuron to neuron synapse	GO:0098984	2.2E-03
Cell projection	GO:0042995	2.8E-03
Plasma membrane region	GO:0098590	3.5E-03
Dendritic spine	GO:0043197	0.013
Plasma membrane	GO:0005886	0.014
Membrane raft	GO:0045121	0.015
Neuron spine	GO:0044309	0.015
Membrane microdomain	GO:0098857	0.015
Postsynaptic membrane	GO:0045211	0.015
Transcription factor*: binding motif (* match class 1)	**Term ID**	**Adjusted *P***
LRF*: GGGGKYNNB	TF:M01100_1	3.3E-06
ZNF37A*: CCYY-GGCTCCNTSCCMN	TF:M12354_1	2.3E-04
ZFP14*: SCNNYCCNGNNSCTSCNC	TF:M12694_1	2.3E-04
CACCC-binding*: CANCCNNWGGGTGDGG	TF:M00721_1	2.6E-04
AP-2alpha*: NGCCYSNNGSN	TF:M01857_1	5.7E-04
RUNX3*: NRACCGCAAACCGCAN	TF:M04109_1	6.9E-04
LRF*: NGKGGGTSNCN	TF:M07387_1	7.0E-04
ZXDL*: GSGSCNNGGGMRGCNCCGGGS	TF:M12722_1	7.0E-04
LUMAN*: CYCAGCYYCY	TF:M09729_1	1.0E-03
HIC1: NNNGGKTGCCCSNNNNNN	TF:M01073	1.1E-03
VDR*: GGGKNARNRRGGWSA	TF:M00444_1	2.1E-03
LRF*: NRGGGKCKY	TF:M10115_1	2.3E-03
ZNF383*: SSNGGGMGGNGSNGGS	TF:M12703_1	2.5E-03
ZGPAT: GRGGCWGNGGNG	TF:M09739	3.4E-03
ZNF614*: NCYCWGCCYYNNN	TF:M09862_1	4.5E-03
MAZ*: GGGGGAGGGGGNGRGRRRGNRG	TF:M09984_1	4.7E-03
Miz-1*: NNRGGWGGGGGAGGGGMRR	TF:M10112_1	7.1E-03
AP-2beta*: GCNNNGGSCNGVGGGN	TF:M01858_1	7.6E-03
GCMa:Erg*: ATGCGGGCGGAARKG	TF:M08487_1	8.0E-03
EGR1*: NNMCGCCCACGCNN	TF:M12508_1	8.3E-03
Elk1:ETV7*: ANSCGGA-CGGATDTCCGGNT	TF:M08214_1	8.3E-03
MOVO-B*: GNGGGGG	TF:M01104_1	9.8E-03
FKLF: BGGGNGGVMD	TF:M01837	0.011
WT1*: SMCNCCNSC	TF:M01118_1	0.011
REST*: NGGCGCTGTCCRTGGTGCTGAA	TF:M12658_1	0.015
ZNF37A: CCYYGGCTCCNTSCCMN	TF:M12354	0.015

Furthermore, we investigated whether ageDMR-associated genes are enriched in known regions of nucleosome retention, based on a previously published data set [27]. There is a significant enrichment of genes associated with mononucleosomes (adj. *P* < 0.001; OR 2.1) as well as with those harboring the histone marks H3K4me3 (adj. *P* = 0.004; OR 1.2) and H3K27me3 (adj. *P* = 0.016; OR 1.2).

### Non-random chromosomal distribution of sperm ageDMRs

**Figure 3:**
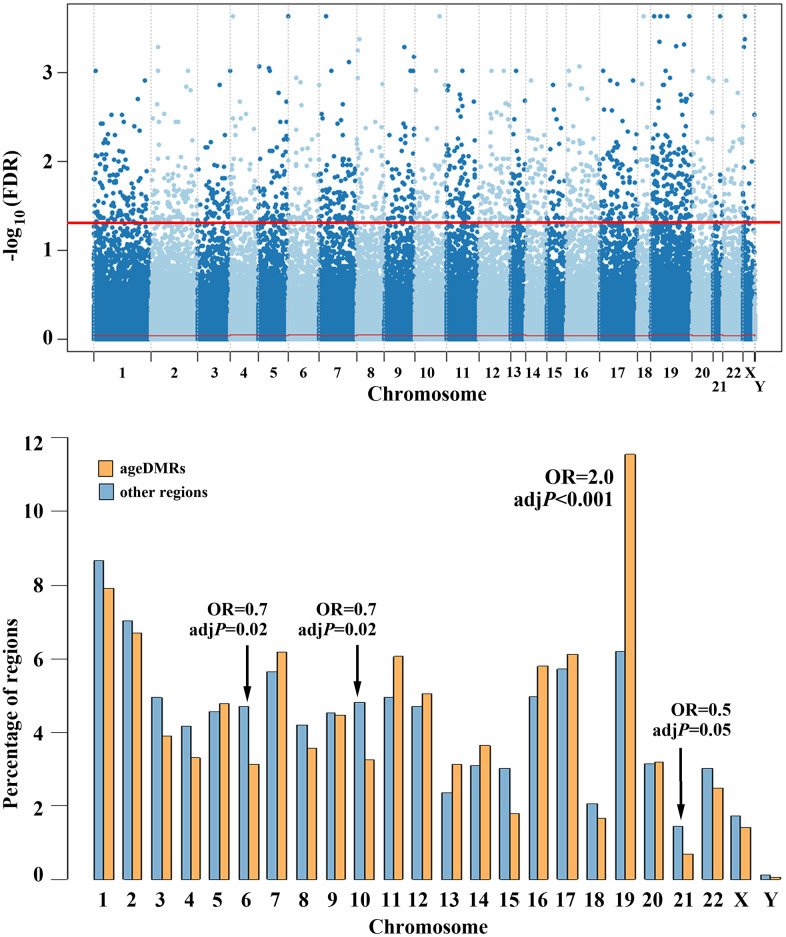
**Chromosomal distribution of human sperm ageDMRs.** The upper panel shows a Manhattan plot of 360,264 regions analysed by RRBS. 1,565 (0.4%) regions above the red line are endowed with genome-wide significant ageDMRs. The bottom plot shows the chromosomal distribution of the 1,565 ageDMRs (orange bars), compared to the 358,699 other (non-significant) regions (blue bars). The y-axis represents the percentage of ageDMRs and other regions, respectively, on each chromosome. AgeDMRs are significantly overrepresented on chromosome 19, and depleted on chromosomes 6, 10, and 21.

The Manhattan plot in Figure 3 (upper diagram) shows the chromosomal distribution of the 360,264 regions, analysed by RRBS. The 1,565 (0.4%) identified sperm ageDMRs are located above the red threshold, indicating genome-wide significant correlation between sperm methylation and donor age. To find out whether or not ageDMRs are randomly distributed throughout the genome, we compared the percentage of ageDMRs on a specific chromosome (Figure 3, bottom, orange bars) to the percentage of other non-significant regions on the same chromosome (blue bars). The percentages of ageDMRs on all chromosomes and the percentages of other regions, respectively, sum up to 100% each. It is striking that chromosome 19 is highly significantly (adjusted *P* < 0.001; OR 2.0) enriched with ageDMRs. It contains 181 (11.6%) of 1,565 ageDMRs, compared to 22,309 (6.2%) of 358,699 non-significant regions. In contrast, ageDMRs are significantly underrepresented on chromosomes 6 (3.1% of all ageDMRs vs. 4.7% of all other regions; adj. *P* = 0.02; OR 0.7), 10 (3.3% vs. 4.8%; adj. *P* = 0.02; OR 0.7), and borderline significant on chromosome 21 (0.7% vs. 1.5%; adj. *P* = 0.05; OR 0.5) (Figure 3, bottom diagram). Interestingly, almost half (245 of 509; 48%) of the zincfinger (ZF) genes that are covered in our RRBS data set are located on chromosome 19. Altogether we identified 14 ageDMRs in 11 ZF genes on chromosome 19, and 14 ageDMRs in 11 ZF genes on other chromosomes. Thus, the ZF genes on chromosome 19 are not enriched with ageDMRs.

To test whether the enrichment of human (*Homo sapiens*, HSA) chromosome 19 with sperm ageDMRs is evolutionarily conserved, we performed RRBS on 11 sperm samples of the New World monkey *Callithrix jacchus* (CJA). Their age ranged from two to 12 years. Without multiple testing adjustment 6,597 of 397,332 analyzed regions were significantly (*P* < 0.01) correlated with donor age, however none of these potential ageDMRs reached genome-wide significance. CJA22 contains 6.4% (25,246 of 397,332) analyzed regions in the marmoset RRBS data set, comparable to HSA19 representing 6.2% (22,490 of 360,264) in the human data set. In contrast to HSA19 which shows a twofold enrichment (11.6%) with sperm ageDMRs, CJA22 was not prone to paternal age effects. Consistent with its size, it contains 6.0% (397 of 6,597) potential sperm ageDMRs. In *Callithrix* none of the chromosomes is enriched or depleted for potential sperm ageDMRs (Supplementary Figure 3).

## DISCUSSION

### Characteristics of sperm ageDMRs

Most previous studies on the aging human sperm methylome have relied on Illumina methylation arrays [15–18]. In addition, methylcytosine capture sequencing [21], RRBS [19], and WGBS [20] have been applied. Differences between techniques (i.e. covered genomic regions), cohorts (i.e. fertile males vs. males undergoing infertility treatment), and sample size may at least partially explain that there is little overlap between ageDMR sets identified in different studies. In most studies [18–21] the hypermethylated DMRs predominate, whereas our study and Jenkins et al.'s [16] show a dramatic excess (74% and 95%, respectively) of hypomethylated DMRs.

As already noted by Denomme et al. [19], the genes with sperm ageDMRs are enriched in regions of developmental importance that have been reported to retain nucleosomes in sperm [27]. However, conflicting evidence suggests that sperm nucleosomes are preferentially located in large gene-poor regions [28, 29].

Consistent with Cao et al. [21], we found that hypomethylated ageDMRs are closer to TSS, the vast majority of them being located in genic regions (TSS; exons and introns). Hypomethylated DMRs are mainly associated with male infertility phenotypes. Methylation array studies [30–32] have already demonstrated an association between sperm methylation changes and fertility status. In this light, it is plausible to assume that hypomethylated sperm ageDMRs mainly reflect the compromized fertility of sperm donors attending a fertility center and may not directly persist in the next generation [33], impacting offspring development.

Almost half of the hypermethylated ageDMRs are located in intergenic regions and the other half in genic regions (mainly introns). Hypermethylated DMRs are enriched in biological processes associated with synapses and in abnormal behavioural phenotypes. Although in our data set they represent only 26% of ageDMRs, these are the primary candidates for mediating paternal age effects on neurodevelopment of the offspring.

### Implications of age-related sperm methylation changes for the next generation

Using RRBS, we identified 1,152 sperm ageDMRs in genic and 413 DMRs in non-genic regions. This is the largest set of candidate genes subject to paternal age effects so far. However, the observed methylation differences between younger and older sperm donors were small, in the order of several percentage points. This is consistent with previous studies on the aging sperm methylome [15–21]. At the level of individual loci (corresponding to ageDMRs), there was considerable overlap in methylation variation between younger and older sperm samples. This supports the view that multiple changes of small effect size rather than highly penetrant epimutations in a single or a few genes contribute to paternal age effects. Sperm aging and the associated medical problems are multifactorial processes, involving genetic and epigenetic changes in numerous genes and pathways. A common approach to interpreting changes in a large number of genes is enrichment analysis.

Including our and the above-mentioned genome-wide studies, altogether 2,355 genes with symbols have been reported with age-related sperm methylation changes so far, however most (90%) of them in only one study. For further analyses, we compiled a list of 241 genes which have been replicated at least once. In this context, it is important to mention that most studies do not provide information on the exact genomic localization (within the gene of interest), effect size, and direction of the observed significant methylation changes. Thus, our meta-analysis is based solely on the symbols of genes reported with sperm ageDMRs. In our RRBS data set, approximately 5% of genes with multiple ageDMRs show methylation changes in opposite direction. Overall, the 241 replicated genes are enriched with a set of transcription factor binding motifs. Consistent with the literature [16, 18–21], they are preferentially associated with the nervous system and synapses. Collectively, our data support the conclusion that age-induced methylation changes in the sperm epigenome contribute to the increased offspring disease susceptibility for neurodevelopmental disorders.

### BMI, fertility status, and reproductive outcome

Our model for sperm methylation analysis included donor age and BMI as numeric parameters as well as semen parameters (normal/abnormal) and pregnancy outcome (yes/no) as categorical parameters. In contrast to the large number of age-related methylation changes, we did not detect DMRs with genome-wide significance for BMI, semen parameters, or pregnancy outcome. Although the absence of genome-wide significant hits does not exclude effects of the paternal BMI or fertility status on the sperm epigenome, it seems plausible to assume that the age effect is much larger than that of BMI and other factors.

Numerous studies [for review, see 12, 34] have postulated a link between sperm methylation changes, in particular in imprinted genes with male infertility. However, because of possible contamination of oligozoospermic samples with cell-free DNA from damaged somatic cells, these results have to be interpreted with caution. All sperm samples in this study were from males attending a fertility center and were purified by two different methods (swim up and density gradient). Samples with severe oligozoospermia were excluded. To detect somatic cell contamination, we performed an in-depth analysis of 8 maternally methylated and three paternally methylated ICRs. Notably, we did not find a single abnormal methylation imprint in a single study sample. Consistent with a recent deep bisulfite sequencing study [26], our results argue against a role for highly penetrant imprinting mutations in male infertility. Although we did not find genome-wide significant DMRs between normal and abnormal spermiograms, functional enrichment analysis showed an association of hypomethylated ageDMRs with male infertility phenotypes. Along with other studies [30–32], this argues in favor of the notion that male infertility has an epigenetic component. Loss of methylation at numerous gene loci with age may contribute to the multifactorial age-related decline of fertility and idiopathic male infertility.

It is generally assumed that the sperm epigenome mediates the effects of paternal factors on embryo development and the next generation [27]. A significant overlap of genes with ageDMRs has been reported between sperm and blastocysts [19]. Other studies [18, 31] have associated age-induced sperm methylation patterns with fertilization, embryo quality, and life birth. To identify predictive markers for male infertility treatment, we compared the methylation patterns of sperm samples which have resulted in a clinical pregnancy and those which have not. However, in our data set there were no genome-wide significant DMRs associated with successful reproductive outcome following ART. Irrespective of semen quality, the baby-take-home rate was higher in the ICSI than in the IVF group.

### Enrichment of sperm ageDMRs on chromosome 19

In our data set, HSA19 showed a highly significant twofold enrichment with sperm ageDMRs, whereas DMRs appeared to be underrepresented on human chromosomes 6, 10, and 21. Previous studies have reported an overrepresentation of age-associated methylation changes on chromosomes 4 and 16 [21] and 19 [19], respectively.

One striking aspect of HSA19 is its high gene density (more than double the genome-wide average) and unusually high GC content (48% compared to 41% genome-wide average) [35]. Moreover, it contains a large number of intrachromosomal segmental duplications (comprising 6.2% of the chromosome), Alu repeats (26% of the chromosome), and ZF transcription factors which are involved in epigenetic repression of endogenous retroviruses and other loci [36]. HSA19 exhibits the highest CpG density of any chromosome in promoter flanking and enhancer regions, consistent with a great regulatory potential by DNA methylation [37]. This may at least partially explain the high susceptibility of HSA19 to paternal age effects on the sperm epigenome.

Although Old World and New World monkeys diverged > 40 million years age [38], the karyotypes of humans and marmosets are surprisingly similar. In particular, the entire HSA19 has been conserved in the orthologous CJA22. In addition, the unusual sequence characteristics of HSA19 have been conserved in non-human primates [36]. Although due to small sample size the identified potential sperm ageDMRs in the marmoset lack genome-wide significance, overall, there was no evidence for a similar enrichment of the HSA19-orthologous CJA22. Potential sperm ageDMRs appeared to be randomly distributed throughout the marmoset genome.

In a candidate gene study [39], we identified three sperm ageDMRs in humans, four in bovine, and three in mice, which were all species-specific. Here, we show a species-specific effect of paternal age on HSA19, which has not been conserved in the marmoset. This supports the idea that sperm ageDMRs are in regions under epigenomic evolution. Species differences in sperm epigenomes may be a driving force to shape lineage-specific complex phenotypes (i.e. brain functioning in humans) to adapt to different environments [40].

## CONCLUSIONS

Using RRBS, we identified > 1,000 candidate genes with genome-wide significant age-related methylation changes in sperm. The majority of the > 1,500 identified sperm ageDMRs became hypomethylated with age and was associated with male infertility phenotypes. The paternal age effect on the next generation may be preferentially driven by hypermethylated sperm ageDMRS which are associated with behavior and neurodevelopment.

One important caveat of our study is that all sperm samples were from males attending a fertility center and, thus, one has to be careful extrapolating results to the normal population. Fertility depends on age, health, and many other factors. It is usually defined as being capable of producing offspring by spontaneous conception. However, even sperm samples from males meeting these criteria may include 5% or more non-paternity events [41]. For an exploratory analysis of the impact of male fertility on the sperm epigenome, we distinguished between samples with normal and abnormal semen parameters. In our final model, paternal age, BMI, semen parameters, and ART outcome were included as factors. In contrast to paternal age, the other factors did not show significant effects. Of course, the fact that we did not observe an effect of semen quality on sperm methylation, does not exclude the existence of such an effect. Indeed, circumstantial evidence from our enrichment analysis suggests an epigenetic component of male infertility.

Despite little overlap between the published gene sets, different genome-wide methylation studies hold a common signal of paternal age. Altogether 241 of 2,355 genes (from 8 studies) with sperm ageDMRs have been replicated and are highly significantly enriched in biological processes and cellular components associated with nervous system development. Circumstantial evidence suggests that the aging sperm epigenome may contribute to the increased disease risk of the offspring of old fathers.

The identified age-associated methylation changes in sperm are numerous, but all are small in effect size within the normal range of methylation variation. Although the underlying mechanism(s) remains unclear, we propose that due to nucleosome retention and/or sperm chromatin packaging loci, sperm ageDMRs are more susceptible to defects in the maintenance of methylation patterns with increasing age. The paternal age effect is not mediated by highly penetrant epimutations in specific, i.e. in imprinted genes but rather is a multifactorial process. Medical problems associated with advanced paternal age may occur when the number of age-induced epigenetic changes and other factors exceeds a critical threshold.

Because of its high gene density and high CpG content chromosome 19 appears to be endowed with a unique regulatory potential by age-related methylation changes. Preliminary evidence suggests that paternal age effects on the sperm epigenome are species-specific and may be part of an evolutionary mechanism(s) for environmental adaptations.

## MATERIALS AND METHODS

### Study samples

The semen samples were collected at the Fertility Center Wiesbaden and written informed consent was obtained from each donor. After IVF/ICSI treatment, the left-over swim-up sperm fraction (excess material) was pseudonymized, and snap-frozen at -80° C until further use. To eliminate contamination by bacteria, lymphocytes, epithelial and other somatic cells, the swim-up sperm samples were gently thawed and purified further by density gradients PureSperm 80 and 40 (Nidacon, Mölndal, Sweden).

Seventy-three sperm samples were used for genome-wide methylation analysis by RRBS: 56 were from men with normal and 17 with abnormal parameters (Supplementary Table 1), using the reference values of the 5^th^ edition of the WHO laboratory manual [24]. Establishment of a pregnancy by IVF or ICSI was determined by biochemical parameters and fetal heart beats. Ninety-four independent sperm samples were used for bisulfite pyrosequencing of candidate genes, the vast majority (92) of them from men with normal spermiograms.

Eleven sperm samples from common marmosets (*Callithrix jacchus*) were obtained by penile vibrostimulation of two- to 12-year old animals housed at the German Primate Center in Göttingen. Swim-up purification of sperm was performed after density gradient purification of fresh sperm samples. Animal experiments were approved by the Niedersächsisches Landesamt für Verbraucherschutz und Lebensmittelsicherheit (no. 42502-04-17/2496).

For DNA isolation, the purified sperm cells were resuspended in 300 μl buffer (5 ml of 5 M NaCl, 5 ml of 1 M Tris-HCl; pH 8, 5 ml of 10% SDS; pH 7.2, 1 ml of 0.5 M EDTA; pH 8, 1 ml of 100% β-mercaptoethanol, and 33 ml of H_2_O), and 100 μl (20 mg/ml; 600 mAU/ml) proteinase K (Qiagen, Hilden, Germany), and incubated for 2 hours at 56° C. Sperm DNA was isolated using the DNeasy Blood and Tissue kit (Qiagen). DNA concentration and purity were measured with a Qubit fluorometer (Thermofisher, Waltham, MA, USA).

### Reduced representation bisulfite sequencing

RRBS enriches for areas of the genome with high CpG content and, therefore, reduces the costs of WGBS by only sequencing a reduced representative subset of the genome, which still contains the majority of promotors and other important genomic features [42]. RRBS libraries were generated from 73 sperm samples (Supplementary Table 1) according to established protocols [43], using the commercially available Ovation RRBS Methyl-Seq System 1-16 (Tecan, Männedorf, Switzerland). Briefly, genomic DNA was digested by the methylation sensitive restriction enzyme MspI, followed by adapter ligation and a final repair step. Then, bisulfite conversion was performed using the EpiTect Fast DNA Bisulfite Kit (Qiagen, Hilden, Germany) for low DNA concentrations. After PCR amplification and bead purification of the final library, DNA concentration was measured with the HS-DNA kit and Qubit fluorometer. Fragment length distribution was assessed by running the samples on the Bioanalyzer 2100 (Agilent, Waldbronn, Germany) using a High Sensitivity DNA Chip. Finally, 2 x 76 paired end sequencing of 16 samples in parallel was performed on the NextSeq500 platform (Illumina) using the NextSeq 500/550 High Output v2 Kit (150 cycles) (Illumina, San Diego, CA, USA).

Following quality control of the sequenced libraries with FastQC (version v0.11.3) adapter trimming has been performed using TrimGalore (version 0.4.0) with subsequent trimming of diversity adapters according to the manufactures protocol and scripts (trimRRBSdiversityAdaptCustomers.py, version 1.11). Next the reads have been mapped to the human reference genome GRCh38 using bismark (version 22.3) with the bowtie2 option activated. Calculation of methylation levels of CpG sites has been performed with methylation extractor of the bismark suite based on the number of reads supporting the methylated and unmethylated state yielding a total of 6,297,704 sites. CpG sites covered in all samples (4,547,455) were subsequently clustered into regions. Adjacent CpGs (with ≤ 70 bp distance between adjacent sites) were aggregated and region-wise beta values derived as weighted average of site-wise methylation values. Only regions with a coverage of five or more in all samples and a non-zero variability across the samples were included in downstream analyses, yielding a total of 360,264 regions after removal of mitochondrial regions. Annotations of regions with genes and transcripts were performed based on the coordinates of the Ensemble gene catalogue provided in the Ensemble package. Genes have been classified as ZF gene, if the corresponding gene symbol starts with the prefix "ZNF". Genic regions have been categorized into regulatory regions around the TSS (including ± 1 kb upstream and downstream), intronic regions, exonic regions, 5’ UTR, 3’ UTR, based on the transcript annotation of the gene catalogue of the Ensembl data base [44]. The remaining regions were regarded as intergenic. The coordinates of imprinting control regions were obtained from a previous study [26].

Association of the mean methylation levels of these regions with age has been analyzed with the linear modelling framework limma including age and BMI as numeric parameters as well as pregnancy (yes/no) and semen parameters (normal/abnormal spermiogram) as categorical parameters in the model. Since BMI information was missing for 9 samples, only 64 samples were included in the final analysis. Multiple testing correction was performed using the Benjamini and Hochberg method [45]. All statistical analyses were performed using R (version 3.2.2) including packages from the Bioconductor project [46].

### Enrichment analyses

Enrichment analysis of hypomethylated and hypermethylated ageDMRs and associated genes was performed with the Genomic Regions Enrichment of Annotations Tool (GREAT) algorithm (http://great.stanford.edu/). The input list contained 1,162 hypomethylated ageDMRs and 403 hypermethylated ageDMRs, respectively, as foreground and all 360,264 analyzed regions covered in our RRBS dataset as background. Foreground/background hypergeometric tests were performed, using default settings (assembly: Hg38, proximal: 5.0 kb upstream, 1.0 kb downstream, plus distal: up to 1000 kb). Briefly, the algorithm links each gene to a regulatory domain in the genome and calculates the total fraction of the genome associated with GO terms, whereby the submitted sites that fall in each annotated GO term region are counted as hits.

The g:Profiler (http://biit.cs.ut.ee/gprofiler/; version: e106_eg53_p16_65fcd97) was used to analyse functional enrichments of the 241 replicated genes, which have been reported to carry sperm ageDMRs in at least two independent studies. The parameters were set as follows: Organism: H. sapiens (hg 38); Input list of genes: ordered query; Data sources Gene Ontology: GO molecular function (GO: MF), GO cellular component (GO: CC), and GO biological process (GO: BP); Data sources biological pathways: KEGG, Reactome, WikiPathways; data sources regulatory motifs in DNA = TRANSFAC, miRTarBase; Data sources protein databases: Human Protein Atlas, Corum; Data sources Human phenotype ontology: HP. The statistical domain scope included only annotated genes. The significance threshold was the g:SCS threshold and the user threshold was set to 0.05.

### Bisulfite pyrosequencing

Polymerase chain reaction (PCR) and sequencing primers (Supplementary Table 6) were designed using the PyroMark Assay Design 2.0 software (Qiagen). DNA methylation standards of 0%, 50%, and 100% methylation were used for assay establishment. PCR for each sample was performed in 25 μl reaction consisting of 2.5 μl 10x PCR buffer with MgCl_2_, 0.5 μl (10 mM) dNTPs, 1.25 μl (10 pmol/ml) of each reverse and forward primer, 0.2 μl (5 U/μl) FastStart Taq DNA polymerase (Roche Diagnostics, Mannheim, Germany), 1 μl (~25 ng) bisulfite converted DNA, and 18.3 μl dH_2_O. PCR amplifications were carried out with an initial denaturation at 95° C for 5 min, 35 cycles of 95° C for 30 s, primer-specific annealing temperature (Supplementary Table 6) for 30 s, and 72° C for 45 s, and a final extension step at 72° C for 10 min. Pyrosequencing was carried out using Pyro Q-CpG software and PyroMark Gold Q96 CDT reagent kit (Qiagen) on the PyroMark Q96 MD system. Unmethylated and fully methylated DNA standards (Qiagen) were used as controls in each pyrosequencing run. In our experience, the average methylation variation between the technical replicates (including bisulfite conversion, PCR, and pyrosequencing) is approximately 1-2 percentage points. Pearson’s correlations were applied to correlate the donor age with the mean DNA methylation level of four selected amplicons, corresponding to the ageDMRs in *PRAM1*, *EEF1A2*, *PRKAR2A*, and *MBD3*. An adjusted *P* value of < 0.05 was considered as statistically significant throughout the analyses.

## Supplementary Material

Supplementary Figures

Supplementary Table 1

Supplementary Table 2

Supplementary Table 3

Supplementary Table 4

Supplementary Tables 5 and 6
