# Supplementary material for: Age-related methylation changes in the human sperm epigenome
Source: Aging (Albany NY). 2023 Feb 27;15(5):1257–78. doi: 10.18632/aging.204546 (PMC10042684; doi:10.18632/aging.204546)
Supplement: Supplementary Figures [file aging-15-204546-s001.pdf]

SUPPLEMENTARY FIGURES

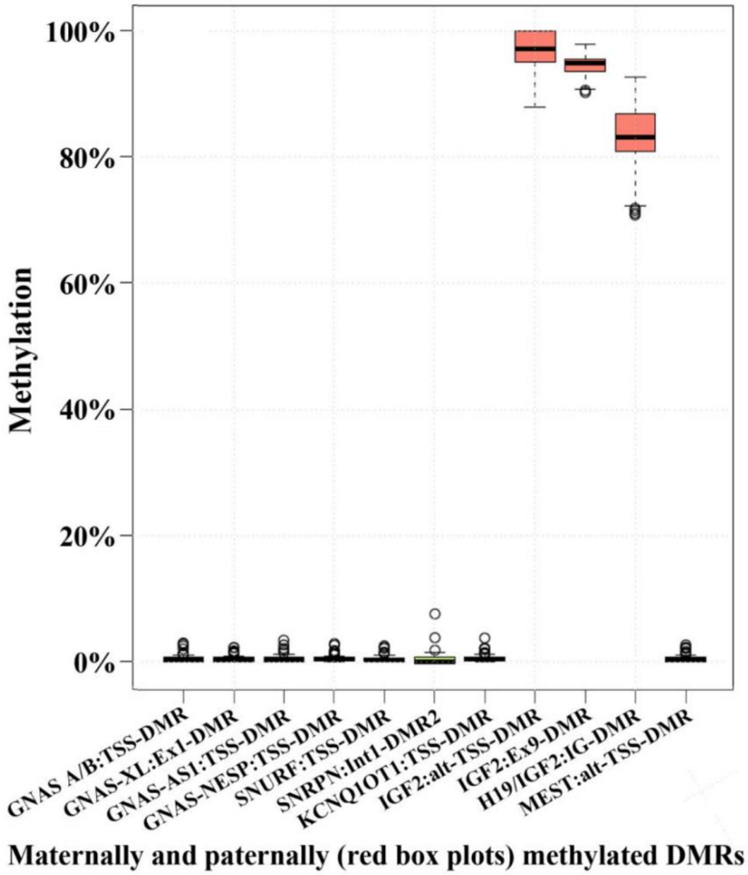

Supplementary Figure 1. Methylation levels of representative imprinted genes. Box plots showing the methylation of 8 maternally and three paternally methylated (red) imprinting control regions (germline DMRs) in our RRBS data set. The median is represented by a horizontal line. The bottom of the box indicates the 25th percentile, the top the 75th percentile. Outliers are shown as circles. All 73 analyzed sperm samples displayed correct methylation patterns of all analysed ICRs.

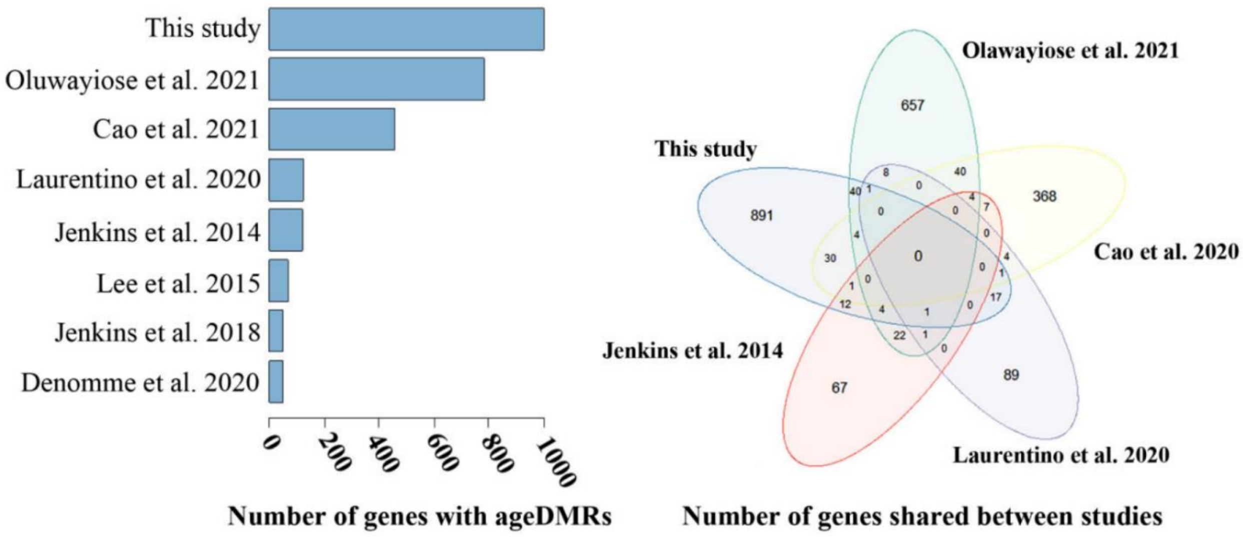

Supplementary Figure 2. Overlap between sperm ageDMRs in different studies. The left bar diagram shows the number of genes with significant sperm ageDMRs identified in 8 independent studies. Denomme et al. [19] listed only imprinted gene. The right Venn diagram shows the number of overlapping gene symbols between the 5 largest studies.

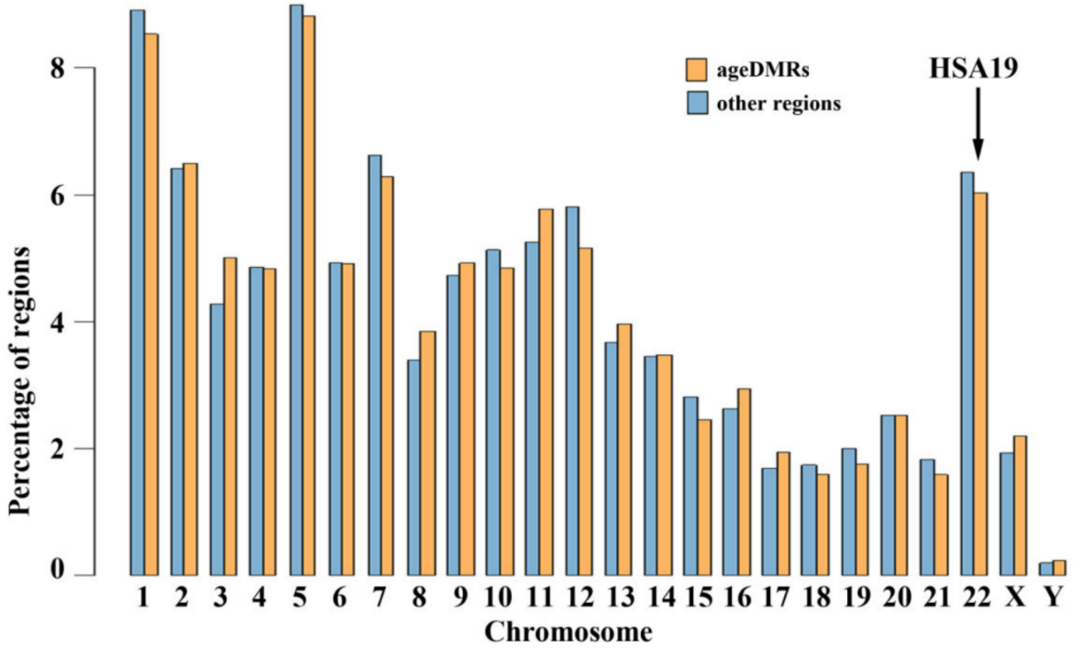

Supplementary Figure 3. Chromosomal distribution of sperm ageDMRs in the marmoset genome. Chromosomal distribution of the 6,597 potential ageDMRs (orange bars) in marmoset sperm, compared to the 390,735 other (non-significant) regions (blue bars). The y-axis represents the percentage of ageDMRs and other regions, respectively, on each CJA chromosome. The percentages of ageDMRs on all chromosomes and the percentages of other regions, respectively, sum up to 100% each.
