# Supplementary material for: Age-related methylation changes in the human sperm epigenome
Source: Aging (Albany NY). 2023 Feb 27;15(5):1257–78. doi: 10.18632/aging.204546 (PMC10042684; doi:10.18632/aging.204546)
Supplement: Supplementary Table 1 [file aging-15-204546-s002.pdf]

SUPPLEMENTARY TABLE

Supplementary Table 1. Clinical parameters of study samples.

Sample ID	Age [years]	Semen parameters*	Concentration [x 10^6/ml]	Normal morphology [%]	Total motility [%]	BMI [kg/m^2]	Infertility treatment	Pregnancy
1	25.8	Abnormal	1	3	16	20.3	ICSI	Yes
2	26.2	Normal	220	12	45	22.7	IVF	No
3	27.9	Normal	136	10	81	31.3	IVF	Yes
4	28.7	Abnormal	1.3	0	1	n.a.	ICSI	Yes
5	29.0	Normal	28	16	63	24.6	IVF	Yes
6	29.0	Abnormal	38	4	50	25.1	IVF	No
7	29.6	Normal	250	15	50	25.4	IVF	No
8	29.7	Abnormal	80	4	60	25.5	IVF	Yes
9	30.3	Normal	83	7	64	18.6	IVF	No
10	30.9	Normal	79	9	58	25.8	IVF	Yes
11	31.4	Normal	85	10	64	24.8	IVF	Yes
12	31.4	Abnormal	13	1	33	n.a.	ICSI	No
13	31.5	Normal	45	5	50	30.4	IVF	No
14	31.8	Normal	55	6	46	28.7	ICSI	No
15	32.7	Normal	50	6	60	27.4	ICSI	No
16	32.9	Normal	95	6	52	31.8	IVF	Yes
17	33.1	Normal	45	7	50	27.4	IVF	No
18	33.1	Abnormal	60	4	44	22.5	IVF	Yes
19	33.3	Normal	81	15	50	n.a.	IVF	No
20	33.4	Normal	68	10	58	n.a.	IVF	Yes
21	34.4	Normal	75	8	64	37.8	IVF	No
22	35.0	Normal	100	6	65	23.8	IVF	No
23	35.1	Normal	91	12	67	26.8	IVF	Yes
24	35.3	Abnormal	6	2	26	23.4	ICSI	Yes
25	35.4	Normal	92	8	60	24.2	ICSI	Yes
26	35.4	Normal	100	6	60	21.6	IVF	No
27	35.7	Normal	65	9	59	23.1	IVF	No
28	35.8	Normal	63	11	65	n.a.	IVF	Yes
29	36.7	Abnormal	14	2	3	24.9	ICSI	Yes
30	37.1	Normal	88	6	47	26.3	IVF	Yes
31	37.3	Normal	187	12	71	17.5	IVF	No
32	37.5	Normal	110	7	64	29.9	IVF	Yes
33	37.5	Normal	46	6	61	30.0	IVF	No
34	38.1	Normal	90	6	60	31.1	ICSI	Yes
35	38.2	Normal	240	8	59	24.6	IVF	No
36	38.5	Normal	88	12	72	25.3	ICSI	Yes
37	38.7	Abnormal	2.4	1	12	27.8	ICSI	Yes
38	38.9	Normal	170	6	55	23.7	IVF	Yes
39	38.9	Normal	45	9	77	30.6	IVF	No
40	39.3	Abnormal	7	2	29	n.a.	ICSI	Yes
41	39.5	Normal	180	10	50	28.3	IVF	Yes

42	39.6	Normal	90	6	75	38.7	IVF	No
43	39.7	Normal	82	12	66	22.0	IVF	Yes
44	39.7	Normal	75	9	67	27.0	IVF	No
45	40.4	Normal	150	6	65	27.4	IVF	Yes
46	40.5	Normal	31	5	42	21.2	IVF	No
47	40.8	Normal	50	5	65	23.5	ICSI	Yes
48	41.1	Normal	80	12	55	n.a.	IVF	No
49	41.2	Normal	80	5	50	24.8	IVF	Yes
50	42.5	Abnormal	15	12	56	30.2	IVF	No
51	42.6	Abnormal	5.1	1	33	22.7	ICSI	Yes
52	42.8	Normal	60	11	55	24.8	IVF	Yes
53	42.9	Normal	82	10	66	28.4	IVF	Yes
54	43.0	Normal	80	6	60	25.7	IVF	No
55	43.2	Normal	110	11	73	27.4	IVF	Yes
56	43.2	Normal	70	15	60	28.7	IVF	No
57	43.3	Abnormal	0.7	2	5	29.3	ICSI	No
58	43.7	Abnormal	17	4	71	25.7	ICSI	No
59	43.7	Normal	40	12	85	25.1	ICSI	No
60	44.1	Normal	200	15	67	27.8	IVF	Yes
61	44.4	Abnormal	14	1	29	26.0	ICSI	No
62	45.2	Normal	65	9	53	25.2	IVF	Yes
63	45.6	Normal	120	12	55	23.4	IVF	Yes
64	45.9	Normal	64	8	64	33.1	IVF	Yes
65	45.9	Normal	59	11	56	n.a.	IVF	No
66	46.0	Normal	91	12	67	28.4	IVF	Yes
67	46.0	Normal	52	10	84	24.4	IVF	No
68	46.3	Normal	35	12	51	23.4	IVF	Yes
69	47.4	Normal	71	14	72	30.4	IVF	Yes
70	48.0	Abnormal	10.6	3	3	n.a.	ICSI	Yes
71	49.0	Normal	100	12	50	22.7	IVF	Yes
72	49.5	Abnormal	7	3	21	22.6	ICSI	Yes
73	50.4	Normal	19	8	59	23.2	IVF	Yes

*According to the WHO laboratory manual, 5th edition, concentration > $15x10^6$ sperm per ml, > 40% motile sperm, and > 4% sperm with normal morphology were considered as normal.
