# Supplementary material for: Age-related methylation changes in the human sperm epigenome
Source: Aging (Albany NY). 2023 Feb 27;15(5):1257–78. doi: 10.18632/aging.204546 (PMC10042684; doi:10.18632/aging.204546)
Supplement: Supplementary Table 3 [file aging-15-204546-s004.pdf]

Supplementary Table 3. Correlations between sperm donor age and mean methylation of selected ageDMRs.

Gene	DMR location	Function	Bisulfite pyrosequencing		
			Methylation (%) mean±SD [range]	Pearson's correlation	FDR-adjusted *P* value*
EEF1A2	Promoter	Encodes an isoform of the alpha subunit of the elongation factor 1 complex. Plays an essential role in protein synthesis by transporting aminoacyl-tRNA to the A-site of the ribosome. Expressed in brain, heart, and skeletal muscle.	14.7±7.7 [1.9-41.6]	-0.23	0.04
MBD3	Promoter	Encodes methyl-CpG-binding domain protein 3. Involved in nucleosome remodeling, transcriptional repression, and histone deacetylase activities. Helps to maintain pluripotency of embryonic stem cells.	55.3±8.7 [34.3-77.6]	-0.25	0.03
PRAM1	Protein coding	Encodes PML-RARA-regulated adapter molecule 1. Expressed and regulated during normal myelopoiesis. Maybe involved in lipid binding, myeloid differentiation, and integrin signaling in neutrophils.	61.4±9.3 [32.3-79.1]	-0.22	0.04
PRKAR2A	Promoter	Encodes cAMP-dependent protein kinase type II-alpha regulatory subunit. Involved in cAMP signaling in cells. Regulates protein transport from endosomes to the Golgi apparatus and further to the endoplasmic reticulum.	21.2±5.3 [8.0-36.7]	-0.34	0.001

*using the Benjamini-Hochberg method.
