# Supplementary material for: Age-related methylation changes in the human sperm epigenome
Source: Aging (Albany NY). 2023 Feb 27;15(5):1257–78. doi: 10.18632/aging.204546 (PMC10042684; doi:10.18632/aging.204546)
Supplement: Supplementary Tables 5 and 6 [file aging-15-204546-s006.pdf]

Supplementary Table 5. Enrichment of the 1,002 ageDMR-associated gene symbols in published studies.

Study	Odds ratio	*P* value	Adjusted *P* value
Jenkins et al., 2014	4.72	4.33E-07	1.16E-06
Lee et al., 2015	6.04	1.65E-06	3.30E-06
Jenkins et al., 2018	5.03	0.002	0.002
Cao et al., 2020	2.14	7.68E-05	8.78E-05
Denomme et al., 2020*	6.68	1.23E-05	1.64E-05
Laurentino et al., 2020	4.76	9.10E-08	3.64E-07
Oluwayiose et al., 2021	2.15	3.56E-06	5.69E-06

*includes only imprinted genes.

Supplementary Table 6. PCR and sequencing primers for bisulfite pyrosequencing of candidate genes with sperm ageDMRs.

Gene	Primer	Sequence (5'-3')[a]	Location[b]	Annealing temp. (° C)	Number of CpGs
EEF1A2	Forward	*GGGGAGAGATGGTTATTGTTTTTTA	Chr 20: 62,129,630-62,129,882	58° C	
	Reverse	CTCCACCTAACACTTACTAAACT			
	Sequencing 1	ACTATTCTAAATTCCTAATCTAAAC			3
	Sequencing 2	AAACCCCCACCTCCC			3
MBD3	Forward	GGAGTTTGAGATTAGGTTGATTTAATAT	Chr 19: 1,583,739-1,584,048	58° C	
	Reverse	*ACAAACATCCACACCTCATAA			
	Sequencing 1	GAGGTAGGAGAATTATTTGA			2
	Sequencing 2	GATGATATTATTGTATTTTTGTTTG			3
PRAM1	Forward	AGGTTGGGAGAATTTTTTTAGTTTATTA	Chr 19: 8,564,542-8,564,724	60° C	
	Reverse	*ATCCTTCCATACCCCTTCTATATATT			
	Sequencing	TGGAATTTTGTTTTGATGT			2
PRKAR2A	Forward	AGTGGTATGATTTTGGTTTATTGTAA	Chr 3: 48,883,704-48,883,995	61° C	
	Reverse	*CTAAACAAAATAAACACCCTACCTC			
	Sequencing 1	AGTGTTGGGATTATAGG			3
	Sequencing 2	GGTTAATTTTTGTATTTTTAGTAGA			1
	Sequencing 3	TTTTTTAAGTAGTTGGGATTATAGA			4

[a]Primers indicated by a star are biotinylated at the 5' end.
[b]Genome Reference Consortium Human Build 37 (GRCh37)/hg37 was used as references.
